# Global, Regional, and Country-Level Economic Impacts of Oral Conditions in 2019

**DOI:** 10.1177/00220345241281698

**Published:** 2024-11-13

**Authors:** M. Jevdjevic, S. Listl

**Affiliations:** 1 Heidelberg University Hospital, Heidelberg Institute of Global Health, Section for Oral Health, Germany

**Keywords:** dental economics, expenditures, productivity, oral health, health services research, health resources

## Abstract

The recent World Health Organization (WHO) Oral Health Resolution and the subsequent WHO Global Oral Health Action Plan highlight the key relevance of providing information on the economic impacts of oral conditions. The purpose of this study was to provide updated estimates for the global, regional, and country-level economic impacts of oral conditions in 2019. Extending previously established methods, dental expenditures (costs for treatments) and productivity losses for 5 oral conditions (caries in deciduous and permanent teeth, periodontitis, edentulism, other oral diseases) were estimated for the year 2019. The estimated total worldwide economic impacts of oral conditions in 2019 were US $710B, of which US $387B (US $327B to US $404B) was due to direct costs and US $323B (US $186 to US $460) was due to productivity losses for the 5 main oral conditions. Low-income countries spent an average of US $0.52 (US $0.22 to US $0.96) per capita on dental care, while high-income countries spent an average of US $260 (US $257 to US $268) per capita—a 500-fold difference. These findings suggest that oral conditions continue to substantiate an enormous economic burden to individuals and society. The comprehensiveness of estimates supersedes that of previous work as the primary information on direct costs was identified for a larger number of countries. The need for more and better routine reporting and monitoring of the economic impact of oral conditions is emphasized. The relevance of such information is also highlighted by its inclusion in the first-ever WHO Global Oral Health Status Report and Global Strategy on Oral health 2023 to 2030. Given the persistently high economic burden of oral conditions, there is a key role for better prioritization of cost-efficient oral health programs as well as needs-responsive capacity planning.

## Introduction

The recent World Health Organization (WHO) Oral Health Resolution, the WHO Oral Health Status Report, and the WHO Oral Health Action Plan highlight the utmost relevance of monitoring the economic impacts of oral conditions to enable access to quality oral health care for everyone without causing financial hardship (WHO 2021, 2022, 2023). Given that available resources to societies are limited, decision makers within and outside health care need to carefully consider how resources should be used to maximize people’s well-being (Listl et al. 2019; Listl et al. 2021). Oral conditions (dental caries, periodontal diseases, tooth loss, oral/pharyngeal cancers) affect more than 3.5 billion people worldwide (Peres et al. 2019) and are the third most expensive diseases to treat in the European Union (Listl et al. 2019). However, 87% of dental expenditures benefit only 22% of the world’s population (Righolt et al. 2018).

While the relevance of monitoring the economic impacts of oral conditions is clear, there continues to be major room for improvement in the regularity and comprehensiveness of reporting the economic impacts of oral conditions globally. While the available information on economic impacts has been improving during recent years, relevant information about country-level representative dental expenditures (= direct costs) was identifiable for only 66 countries when making estimates for 2010 (Listl et al. 2015) and still only 73 countries when making estimates for 2015 (Righolt et al. 2018). The limited reporting of dental expenditure data has also been shown to complicate the forecasting of future dental expenditures (Jevdjevic et al. 2021). In addition, estimates of productivity losses (= indirect costs) have been dependent on disability-adjusted life-year (DALY) statistics and their associated limitations (Listl et al. 2015; Righolt et al. 2018). Hence, cyclical updating and improvement of transparent information about the economic impacts of oral conditions remain essential for ensuring that oral health systems become more efficient and more equitable.

Against this background, the purpose of this study was to provide an update of estimates of the global, regional, and country-level dental expenditures as well as productivity losses relating to oral conditions in the year 2019. The year 2019 was deliberately chosen because this represents the relevant reporting year for the WHO Global Oral Health Status Report (WHO 2022), and the present study was designed to contribute economic insights for this report. The comprehensiveness of estimates supersedes that of previous work by incorporating primary information on direct costs from a greater number of countries and relying on more comprehensive data. In addition, they provide further insights into underlying uncertainties. Note that careful consideration is needed when comparing the 2019 estimates with those from 2010 and 2015.

## Methods

A previously established methodological framework (Listl et al. 2015; Righolt et al. 2018) was used to estimate global- and country-level direct and indirect costs of oral conditions in the year 2019. Direct costs were defined as overall expenditures for dental health care (including public and private expenditures). Indirect costs aim to account for decreased or lost productivity associated with the burden of 5 main oral conditions (caries of deciduous teeth, caries of permanent teeth, chronic periodontitis, edentulism [total tooth loss], and other disorders [a heterogeneous group including a variety of tooth, tongue, and jaw disorders and malformations not included in the other causes]). For instance, decreased productivity reflects the financial losses industries face when working individuals have compromised working capacity due to severe toothache or side effects of dental treatments. Lost productivity includes the time spent receiving a dental treatment (e.g., workforce sick leaves and absenteeism of parents or caregivers from work).

To complement the information on oral health status for the WHO Global Oral Health Status Report (WHO 2022), the United Nations (UN)/WHO categorization of countries was applied.

### Estimation of Direct Costs

#### Selection of studies

To identify country-specific yearly national expenditures for outpatient dental care in 2019 or nearest year available, electronic searches were performed within the following online resources: WHO Global Health Expenditure Database (https://apps.who.int/nha/database) OECD Data (https://data.oecd.org/), FDI Oral Health Atlas (http://issuu.com/myriadeditions/docs/flipbook_oral_health), Platform for Better Oral Health in Europe (www.oralhealthplatform.eu), Council of European Chief Dental Officers (http://www.cecdo.org/), Intergovernmental Organization Search (http://www.uia.org/igosearch), and noncustomized Google search (www.google.com) in English, French, and Spanish. Thereby, search terms for expenditure (“expenditure,” “expenditures,” “cost”, “costs,” or “treatment costs”) were combined with search terms for dental care (“dentist,” “dental,” “dentistry,” “oral health,” “oral health care,” “oral health services,” or “dental care”). The “[country name]” was used as an additional search term for each country. Further, governmental websites on health and health financing (e.g., Ministry of Health webpages) were explored with a particular focus on any file or report related to the National Health Accounts.

We also searched MEDLINE via PubMed (keyword- and MeSH-based searches), EMBASE via OVID, LILACS via BIREME, the Cochrane Database of Systematic Reviews, the Database of Abstracts of Reviews of Effects, the Health Technology Assessment Database, and the NHS Economic Evaluation Database. In addition, bibliographies of publications identified to be relevant were hand searched. To be eligible, information sources had to report the overall annual national expenditures (public and private source of funds) on dental health care for a specific country, such as specified in the International Classification of Health Accounts (categories: HC.1.3.2 “Outpatient dental care,” HP.3.2 “Offices of dentists”; OECD 2000). Given the dearth of relevant information for the year 2019, identifiable data were located for the year 2018 (or earlier years after 2000) and then extrapolated to 2019. We included the expenditures expressed as a percentage of gross domestic product (GDP) or absolute monetary values. Information sources were excluded if they were considered to report incomplete or unclear information about dental expenditures (e.g., only private out-of-pocket payments; expenditures for a nonrepresentative population group). One author (M.J.) independently carried out all searches and screened the information sources according to the inclusion and exclusion criteria. Searching and screening were independently rerun and double checked by the other author (S.L.). In cases in which exclusion or inclusion of information was unclear, a final decision was made by consensus among the authors.

#### Data extraction and imputation

We computed global and country-specific dental expenditures in 2019 US dollar values using country-specific information obtained from the literature search (see the above strategy). We converted costs reported in local currencies using mid-year conversion rates for the reporting year (http://www.xe.com) and adjusting for inflation rates as well as purchasing power parity (http://www.usinflationcalculator.com). If expenditures were originally reported as percentage of GDP, it was multiplied by the respective country’s population size and 2019 GDP per capita (nominal). GDP per capita (nominal) and population size values were extracted from the World Economic Outlook Database (International Monetary Fund 2024). The information on GDP was missing for Cook Islands, Monaco, and Niue. Further, missing information in the World Economic Outlook Database (population sizes for Andorra, North Korea, Cuba, Egypt, Sao Tome and Principe, Sudan, Syria) was complemented by information from the World Bank Open Data (World Bank 2024b). For countries with no identifiable relevant information, cost estimates were imputed, using the mean dental expenditure (in percentage of GDP) of the nearest geographic unit with available data and multiplying it with the GDP value of the respective country. To estimate uncertainty intervals for the countries with missing information, the lowest dental spending in terms of percentage GDP of the nearest geographic unit with available data (lower bound) and the highest dental spending as percentage GDP of the nearest geographic unit with available data (upper bound) were multiplied with the GDP value of the respective country with missing information. If the information was not available within the same subregion, the estimates from the nearest region were used as defined by the Global Burden of Disease (GBD) study.

### Estimation of Indirect Costs

Drawing from an approach developed by the WHO’s Commission on Macroeconomics and Health and previously applied to estimate indirect costs of oral conditions (WHO 2001; Listl et al. 2015; Righolt et al. 2018), country-specific GDP per capita values (nominal) from the World Economic Outlook Database (International Monetary Fund 2024) were multiplied with DALY estimates for the 5 oral conditions as reported by the GBD 2019 Study (GBD 2019 Diseases and Injuries Collaborators. 2020; Jevdjevic & Listl 2022): caries of deciduous teeth, caries of permanent teeth, chronic periodontitis, edentulism (total tooth loss), and other disorders (a heterogeneous group including a variety of tooth, tongue, and jaw disorders and malformations not included in the other causes, excluding orofacial clefts and lip and oral cavity cancer). A DALY represents a time-based metric that allows a comparison of the burden of various health conditions. It accounts for years of life lost to due to premature mortality and the years lived with a disability due to a specific health condition. By factoring in the country-specific value of a DALY with the GDP per capita, we estimated the economic output lost due to disability caused by oral conditions (WHO 2001). Missing information in the World Economic Outlook Database (see above) was complemented by information from World Bank Open Data (World Bank 2024b).

For the global, regional, and country level, we computed total aggregate and per capita estimates of direct and indirect costs due to the oral conditions defined above. Estimates were also aggregated by WHO Regions (African Region, Eastern Mediterranean Region, European Region, Region of the Americas, South-East Asia Region, Western Pacific Region) and by World Bank Income Groups for countries (low income, lower middle income, World Bank upper middle income, World Bank high income). Calculations were performed with Microsoft Excel v.12.0.6765.5000.

## Results

### Direct Costs

The country-specific electronic searches identified relevant information for 98 of 194 countries. Cost estimates were imputed for 93 (48%) countries with missing data. The estimated worldwide expenditures due to oral conditions in 2019 and by WHO regions as well as World Bank income country groups are presented in Table 1. Overall, the direct treatment cost amounted to US $387.09B (US $327.43 to US $404.38). Across WHO regions, the highest aggregate levels of expenditures were found for the region of the Americas (US $156.76B), the European Region (US $112.51B), and the Western Pacific Region (US $107.00B). Lower expenditures were observed for the Eastern Mediterranean Region ($6.97B), the African Region ($3.10B), and the South-East Asia Region ($0.76B). The average per capita dental expenditure in low-income countries amounted to $259.96 in World Bank high-income countries compared with $0.52 in World Bank low-income countries. Of the dental expenditures, 78% occurred in World Bank high-income countries, 21% in World Bank upper-middle-income countries, and the remaining 1% of expenditures occurred in the World Bank lower-middle-income and low-income countries. Country-level estimates for expenditures due to oral conditions are shown in Table A.1 (Appendix). The highest expenditures were found for the United States ($133.51B), followed by China ($61.55B), Germany ($30.88B), Japan ($28.73B), Italy ($19.12B), and Canada ($12.83B). Most countries with primary data available were found to spend less than 0.5% of the national GDP on dental treatment costs. The highest proportion of dental treatment costs relative to national GDP was found for Armenia and Italy, each with dental treatment costs at approximately 1.0% relative to the national GDP. The lowest proportions were found for Iraq and Kenya, each with dental treatment costs of less than 0.000001% relative to national GDP.

**Table 1.** Direct Costs (Treatment Expenditures) due to oral conditions in 2019 by World Regions.

	Direct Costs		
Region	Total ($US, billions) with Uncertainty Intervals	Per Capita ($US), with Uncertainty Intervals	Proportion of Countries with Available Information (%)
WHO African region	3.10 (2.46–5.17)	2.84 (2.46–4.73)	38.30
WHO Eastern Mediterranean region	6.97 (4.37–15.86)	9.78 (6.14–22.27)	42.86
WHO European region	112.51 (111.76–113.87)	120.96 (120.15–122.42)	81.13
WHO region of the Americas	156.76 (154.58–160.69)	155.21 (153.05–159.10)	22.86
WHO South-East Asia region	0.76 (0.41–0.97)	0.38 (0.21–0.49)	72.72
WHO Western Pacific region	107.00 (53.85–107.82)	54.74 (27.55–55.16)	56.00
World Bank low income	0.36 (0.16–0.67)	0.52 (0.22–0.96)	22.58
World Bank lower middle income	2.27 (1.31–4.41)	0.72 (0.42–1.40)	47.27
World Bank upper middle income	80.76 (25.82–86.49)	29.99 (9.59–32.11)	45.45
World Bank high income	303.70 (300.15–312.81)	259.96 (256.93–267.77)	72.73
Total	387.09 (327.43–404.38)	50.27 (42.52–52.51)	52.08

Data source: Jevdjevic M, Listl S, “Economic impacts of oral diseases in 2019 - data for 194 countries” [database], Heidelberg Open Research Data (heiDATA), 2022. https://doi.org/10.11588/data/JGJKK0.

### Indirect Cost

Table 2 shows a total estimated global indirect cost of US $322.69B (US $185.60 to US $459.78) due to the 5 main oral conditions in 2019. Across WHO regions, the highest aggregate levels of productivity losses were found for the Americas ($105.57B), the European Region ($104.48B), and the Western Pacific Region ($85.12B). Lower productivity losses were observed for the South-East Asia region ($13.35B), the Eastern Mediterranean region ($9.59B), and the African region ($4.58B). Of the productivity losses, 67% occurred in the World Bank high-income countries, 27% in the World Bank upper-middle-income countries, and the remaining 6% in the World Bank lower-middle-income and low-income countries. Country-level estimates for expenditures due to the 5 main oral conditions are shown in Table A.2 (Appendix). The highest productivity losses due to the 5 main oral conditions were found for the United States ($78.47B), followed by China ($45.71B), Japan ($23.66B), Germany ($19.40B), France ($11.99B), and the United Kingdom ($11.20B). Across all countries, $1.55B of productivity losses were attributable to caries in deciduous teeth, $22.46B to caries in permanent teeth, $82.10 to periodontitis, $167.29B to edentulism, and $49.28B to other oral conditions.

**Table 2.** Indirect Costs (Productivity Losses) due to 5 Main Oral Conditions in 2019.

	Indirect Costs (95% UI)	
Oral Condition	Total ($US, billions)	Per Capita ($US)
Caries of deciduous teeth	1.55 (0.00–3.50)	0.20 (0.00–0.45)
Caries of permanent teeth	22.46 (5.38–39.54)	2.92 (0.70–5.13)
Periodontal diseases	82.10 (8.83–155.37)	10.66 (1.15–20.18)
Edentulism	167.29 (120.92–213.66)	21.72 (15.70–27.75)
Other oral disorders	49.28 (29.00–69.56)	6.40 (3.77–9.03)
Total	322.69 (185.60–459.78)	41.90 (24.10–59.71)

Main data source: Jevdjevic M, Listl S, “Economic impacts of oral diseases in 2019 - data for 194 countries,” Heidelberg Open Research Data (heiDATA), 2022, https://doi.org/10.11588/data/JGJKK0. Uncertainty intervals (UIs) were derived from 95% UI ranges for condition-specific disability-adjusted life-years in 2019 (GBD 2019 Diseases and Injuries Collaborators 2020).

## Discussion

The findings of the present study indicate a global economic burden of $710B due to oral conditions in 2019, of which $387B was due to direct costs and $323B was due to productivity losses for the 5 main oral conditions. Low-income countries were found to spend an average of $0.52 per capita on dental care, whereas high-income countries spent an average of $260 per capita—a 500-fold difference. These findings suggest that oral conditions continue to substantiate an enormous economic burden to individuals and society.

In comparison with previously reported data (Listl et al. 2015; Righolt et al. 2018), the data presented here suggest that the total economic burden of oral conditions in 2019 was larger than that for 2010 and 2015. For 2010, direct costs were estimated at $298B and indirect costs at $144B (Listl et al. 2015). For 2015, direct costs were estimated at $357B and indirect costs at $188B (Righolt et al. 2018). Considering general trends in worldwide health spending since 2010 (GBD 2021 Health Financing Collaborator Network 2023; World Bank 2024a), direct costs (dental expenditures) remained stable at a level of 4.6% relative to overall health spending.

The strengths of this study include its high policy relevance, the inclusion of best-available data for the year 2019, and the use of previously established methodologies to provide updated estimates of the global, regional, and country-level economic impacts of oral conditions. Previous work on the economic impact of oral conditions has been instrumental for raising awareness and better prioritization of oral health in global policy making (WHO 2024a). The relevance of the data underlying this study has been referenced and highlighted by WHO’s first-ever Global Oral Health Status Report, in the WHO Global Health Observatory, and by the World Economic Forum (WHO 2022, 2024b, World Economic Forum 2024). Understanding the economic impacts of oral conditions is essential for evidence-informed oral health policy making, including for the prioritization of cost-efficient oral health programs and needs-based planning of the oral health workforce (Listl et al. 2023). Another strength of the present study is its use of hitherto unavailable comprehensive input data. While relevant information about country-level representative dental expenditures (= direct costs) was identifiable for only 66 countries when making estimates for 2010 (Listl et al. 2015) and for only 73 countries when making estimates for 2015 (Righolt et al. 2018), the present study’s estimates of dental expenditures are based on country-level representative data for 98 of 194 countries, which is a major improvement compared with previous studies. Drawing from previously established methodology, the findings of the present study also enable relevant comparisons with previous studies on the economic impacts of oral conditions (WHO 2001; Listl et al. 2015; Righolt et al. 2018).

The present study is not without limitations. In particular, there is still room for improvement in the availability and harmonized reporting of data to support the estimation of economic impacts due to oral conditions. For nearly 50% of countries, no relevant country-level information on dental expenditures could be identified, and these countries’ direct costs had to be approximated by imputation. Moreover, the use of oral condition–related DALY categories from the GBD study has been evolving over time. While previous estimates of indirect costs due to oral conditions were based on DALYs for caries, periodontitis, and edentulism, the present study draws from the GBD study’s 2019 reporting of DALY estimates for 5 oral conditions (caries of deciduous teeth, caries of permanent teeth, chronic periodontitis, edentulism, and other oral disorders). To enable comparability of 2019 productivity loss estimates with estimates for 2015 and 2010, the present study also includes productivity loss estimates specifically for caries, periodontitis, and tooth loss. For meaningful comparisons of economic impacts of oral conditions over time, the relevance of harmonized reporting of underlying data is crucial. Note that the present study followed the UN/WHO country categorization to support harmonized reporting in the WHO Global Oral Health Status Report (WHO 2022). Future work on the worldwide economic impact of oral conditions might benefit from further improvements in the availability of county-level reports on dental treatment costs, harmonized reporting of dental expenditures by oral conditions, evaluation of trends in economic impacts by means of a consistent methodological framework, and addressing the various sources of uncertainties, which still persist in estimating economic impacts of oral conditions.

## Conclusion

The present article provides updated estimates for global and country-level economic impacts of oral conditions. Within the limitations of available data sources, the economic impact of oral conditions amounted to $710B in 2019, of which US $387B was due to direct costs and US $323B was due to productivity losses for the 5 main oral conditions. These findings suggest that oral conditions continue to substantiate an enormous economic burden to individuals and society. The need for more and better routine reporting and monitoring of the economic impact of oral conditions is emphasized. Given the persistently high economic burden of oral conditions, there is a key role for better prioritization of cost-efficient oral health programs as well as needs-responsive capacity planning.

## Author Contributions

M. Jevdjevic, S. Listl, contributed to conception and design, data acquisition, analysis, and interpretation, drafted and critically revised the manuscript. All authors gave final approval and agree to be accountable for all aspects of the work.

## Supplemental Material

sj-docx-1-jdr-10.1177_00220345241281698 – Supplemental material for Global, Regional, and Country-Level Economic Impacts of Oral Conditions in 2019 Supplemental material, sj-docx-1-jdr-10.1177_00220345241281698 for Global, Regional, and Country-Level Economic Impacts of Oral Conditions in 2019 by M. Jevdjevic and S. Listl in Journal of Dental Research
