# Supplementary material for: Global, Regional, and Country-Level Economic Impacts of Oral Conditions in 2019
Source: J Dent Res. 2024 Nov 13;104(1):17–21. doi: 10.1177/00220345241281698 (PMC11662506; doi:10.1177/00220345241281698)
Supplement: sj-docx-1-jdr-10.1177_00220345241281698 – Supplemental material for Global, Regional, and Country-Level Economic Impacts of Oral Conditions in 2019 [file sj-docx-1-jdr-10.1177_00220345241281698.docx]

**Appendix**

**Table A.1:** Country-level direct costs (treatment expenditures) due to dental diseases in 2019

| ***Country*** | ***Total ($US, bn)*** | ***Per capita ($US)*** | ***Notes*** |
| --- | --- | --- | --- |
| Afghanistan | 0.0167 (0.0004 – 0.0780) | 0.46 (0.01 – 2.14) | Imputed |
| Albania | 0.0363 (0.0169 – 0.0541) | 12.66 (5.88 – 18.86) | Imputed |
| Algeria | 0.3237 (<0.0001 – 1.4391) | 7.46 (<0.00 – 33.15) | Imputed |
| Andorra | 0.0146 (0.0018 - 0.0174) | 189.89 (23.40 - 225.42) | Imputed |
| Angola | 0.0702 (0.0657 – 0.0746) | 2.33 (2.18 – 2.48) | Imputed |
| Antigua and Barbuda | 0.0048 (0.0017 - 0.0081) | 51.88 (18.15 - 86.59) | Imputed |
| Argentina | 1.1060 (0.7905 – 1.7019) | 24.55 (17.55 – 37.78) | Imputed |
| Armenia | 0.1130 | 38.05 | Primary information available |
| Australia | 7.3703 | 288.25 | Primary information available |
| Austria | 2.1157 | 236.39 | Primary information available |
| Azerbaijan | 0.1268 (0.0153 – 0.4104) | 12.61 (1.52 – 39.90) | Imputed |
| Bahamas | 0.0329 (0.0100 – 0.0479) | 75.30 (26.34 – 125.67) | Imputed |
| Bahrain | 0.0625 (<0.0000 – 0.2778) | 41.36 (<0.00 – 183.83) | Imputed |
| Bangladesh | 0.0125 | 0.08 | Primary information available |
| Barbados | 0.0152 (0.0053 – 0.253) | 52.80 (18.47 – 88.12) | Imputed |
| Belarus | 0.1371 (0.0299 - 0.3063) | 14.47 (3.16 - 32.33) | Imputed |
| Belgium | 2.1221 | 185.20 | Primary information available |
| Belize | 0.0057 (0.0020 – 0.0095) | 14.08 (4.93 – 23.50) | Imputed |
| Benin | 0.0033 (<0.0000 – 0.0186) | 0.28 (<0.00 – 1.58) | Imputed |
| Bhutan | 0.0000 | 0.06 | Primary information available |
| Bolivia (Plurinational State of) | 0.0482 (0.0482 - 0.1037) | 4.17 (4.17 - 8.98) | Imputed |
| Bosnia and Herzegovina | 0.0727 | 20.75 | Primary information available |
| Botswana | 0.0574 (0.0037 - 0.1111) | 24.15 (1.56 - 46.73) | Imputed |
| Brazil | 3.7128 (2.6536 – 5.7134) | 17.68 (12.64 – 27.21) | Imputed |
| Brunei Darussalam | 0.0091 | 20.35 | Primary information available |
| Bulgaria | 0.1094 | 15.72 | Primary information available |
| Burkina Faso | 0.0046 (<0.0000 - 0.0261) | 0.23 (<0.00 - 1.29) | Imputed |
| Burundi | 0.0013 (<0.0000 – 0.0046) | 0.12 (<0.00 – 0.40) | Imputed |
| Cabo Verde | 0.0034 | 6.12 | Primary information available |
| Cambodia | <0.0001 | <0.01 | Primary information available |
| Cameroon | 0.0066 | 0.26 | Primary information available |
| Canada | 12.8291 | 342.52 | Primary information available |
| Central African Republic | 0.0011 (0.0011 – 0.0011) | 0.22 (0.20 – 0.23) | Imputed |
| Chad | 0.0034 (<0.0001 – 0.0193) | 0.26 (<0.00 – 1.51) | Imputed |
| Chile | 0.4696 (0.3356 – 0.7227) | 24.58 (17.57 – 37.82) | Imputed |
| China | 61.5486 (9.4566 – 61.5486) | 43.96 (6.75 - 43.96) | Imputed |
| Colombia | 0.7051 (0.5660 – 0.8442) | 14.00 (11.23 – 16.76) | Imputed |
| Comoros | 0.0003 (<0.0000 – 0.0009) | 0.30 (<0.00 – 1.03) | Imputed |
| Congo | 0.0047 | 1.03 | Primary information available |
| Cook Islands | NA | NA | GDP missing [IMF/World Bank] |
| Costa Rica | 0.1617 | 31.84 | Primary information available |
| Côte d'Ivoire | 0.0066 | 0.25 | Primary information available |
| Croatia | 0.1494 (0.0695 – 0.2227) | 36.80 (17.11 – 54.84) | Imputed |
| Cuba | 0.3044 (0.1065 – 0.5081) | 26.85 (9.39 – 44.81) | Imputed |
| Cyprus | 0.0126 | 14.41 | Primary information available |
| Czechia | 0.8438 | 79.32 | Primary information available |
| Democratic People's Republic of Korea | 0.0895 (0.0138 - 0.0895) | 3.50 (0.54 – 3.50) | Imputed |
| Democratic Republic of the Congo | 0.0229 (0.0214 – 0.0243) | 0.23 (0.22 – 0.25) | Imputed |
| Denmark | 1.8676 | 321.67 | Primary information available |
| Djibouti | 0.0008 (<0.0000 – 0.0029) | 0.76 (<0.00 – 2.64) | Imputed |
| Dominica | 0.0019 (0.0007 – 0.0032) | 26.92 (9.42 – 44.92) | Imputed |
| Dominican Republic | 0.0823 | 7.94 | Primary information available |
| Ecuador | 0.1257 | 7.28 | Primary information available |
| Egypt | 0.3293 | 3.32 | Primary information available |
| El Salvador | 0.0628 (0.0505 – 0.0752) | 9.37 (7.52 – 11.22) | Imputed |
| Equatorial Guinea | 0.0057 (0.0053 – 0.0061) | 4.19 (3.92 – 4.45) | Imputed |
| Eritrea | 0.0024 (<0.0000 – 0.0083) | 0.39 (<0.00 – 1.34) | Imputed |
| Estonia | 0.1490 | 113.00 | Primary information available |
| Eswatini | 0.0138 (0.0009 – 0.0268) | 12.18 (0.79 – 23.56) | Imputed |
| Ethiopia | 0.0001 | <0.01 | Primary information available |
| Fiji | 0.0069 | 7.70 | Primary information available |
| Finland | 0.5937 | 107.59 | Primary information available |
| France | 12.6660 | 195.39 | Primary information available |
| Gabon | 0.0077 | 3.69 | Primary information available |
| Gambia | 0.0000 | 0.02 | Primary information available |
| Georgia | 0.0116 | 3.13 | Primary information available |
| Germany | 30.8773 | 372.15 | Primary information available |
| Ghana | 0.0008 | 0.03 | Primary information available |
| Greece | 0.8265 | 77.14 | Primary information available |
| Grenada | 0.0035 (0.0012 – 0.0058) | 32.04 (11.21 – 53.47) | Imputed |
| Guatemala | 0.1624 (0.1303 – 0.1944) | 9.22 (7.40 – 11.04) | Imputed |
| Guinea | 0.0032 (<0.0000 – 0.0182) | 0.23 (<0.00 – 1.33) | Imputed |
| Guinea-Bissau | 0.0005 (<0.0000 – 0.0026) | 0.25 (<0.00 – 1.44) | Imputed |
| Guyana | 0.0113 (0.0039 - 0.0188) | 14.38 (5.03 - 24.00) | Imputed |
| Haiti | 0.0263 (0.0092 – 0.0439) | 2.34 (0.82 – 3.90) | Imputed |
| Honduras | 0.0520 (0.0417 – 0.0623) | 5.42 (4.35 – 6.49) | Imputed |
| Hungary | 0.3639 | 37.29 | Primary information available |
| Iceland | 0.1455 | 407.54 | Primary information available |
| India | 0.0645 | 0.05 | Primary information available |
| Indonesia | 0.2666 (0.0010 – 0.4842) | 1.00 (<0.01 – 1.81) | Imputed |
| Iran (Islamic Republic of) | 3.5071 | 42.12 | Primary information available |
| Iraq | <0.0001 | <0.01 | Primary information available |
| Ireland | 0.5148 | 104.00 | Primary information available |
| Israel | 1.8272 | 201.81 | Primary information available |
| Italy | 19.1230 | 316.82 | Primary information available |
| Jamaica | 0.0449 (0.0157 – 0.0750) | 15.62 (5.46 – 26.07) | Imputed |
| Japan | 28.7336 | 227.70 | Primary information available |
| Jordan | 0.0747 (<0.0000 – 0.3320) | 7.42 (<0.00 – 32.97) | Imputed |
| Kazakhstan | 0.0610 | 3.27 | Primary information available |
| Kenya | <0.0001 | <0.01 | Primary information available |
| Kiribati | 0.0003 (<0.0000 – 0.0007) | 2.61 (0.28 – 6.04) | Imputed |
| Kuwait | 0.2182 (<0.0000 – 0.9699) | 46.41 (<0.00 – 206.31) | Imputed |
| Kyrgyzstan | 0.0039 | 0.60 | Primary information available |
| Lao People's Democratic Republic | 0.0045 (<0.0001 – 0.0082) | 0.63 (<0.01 – 1.15) | Imputed |
| Latvia | 0.1120 | 58.08 | Primary information available |
| Lebanon | 0.1049 | 17.29 | Primary information available |
| Lesotho | 0.0093 (0.0006 – 0.0181) | 4.56 (0.29 – 8.83) | Imputed |
| Liberia | 0.0007 (<0.0000 – 0.0042) | 0.16 (<0.00 – 0.93) | Imputed |
| Libya | 0.0615 (<0.0000 – 0.2732) | 9.34 (<0.00 – 41.53) | Imputed |
| Lithuania | 0.1990 | 71.47 | Primary information available |
| Luxembourg | 0.2120 | 345.35 | Primary information available |
| Madagascar | 0.0042 (<0.0000 – 0.0145) | 0.15 (<0.00 – 0.53) | Imputed |
| Malawi | 0.0025 (<0.0000 – 0.0086) | 0.12 (<0.00 – 0.42) | Imputed |
| Malaysia | 0.2268 | 6.91 | Primary information available |
| Maldives | 0.0006 | 1.70 | Primary information available |
| Mali | 0.0013 | 0.07 | Primary information available |
| Malta | 0.0444 | 91.64 | Primary information available |
| Marshall Islands | 0.0003 (<0.0001 – 0.0007) | 5.84 (0.63 – 13.51) | Imputed |
| Mauritania | 0.0017 (<0.0000 - 0.0099) | 0.43 (<0.00 - 2.43) | Imputed |
| Mauritius | 0.0168 | 13.26 | Primary information available |
| Mexico | 2.1030 | 16.70 | Primary information available |
| Micronesia (Federated States of) | 0.0005 (<0.0000 – 0.0013) | 5.30 (0.57 – 12.26) | Imputed |
| Monaco | NA | NA | GDP missing [IMF/World Bank] |
| Mongolia | 0.0352 (0.0042 – 0.1112) | 10.65 (1.29 – 33.72) | Imputed |
| Montenegro | 0.0057 | 9.18 | Primary information available |
| Morocco | 0.0821 | 2.31 | Primary information available |
| Mozambique | 0.0049 (<0.0000 – 0.0169) | 0.16 (<0.00 – 0.54) | Imputed |
| Myanmar | 0.0004 | 0.01 | Primary information available |
| Namibia | 0.0028 | 1.13 | Primary information available |
| Nauru | 0.0002 (<0.0000 - <0.0000) | 14.80 (<0.00 – 0.50) | Imputed |
| Nepal | 0.0891 | 3.13 | Primary information available |
| Netherlands | 3.6785 | 213.48 | Primary information available |
| New Zealand | 0.5353 | 106.27 | Primary information available |
| Nicaragua | 0.0314 (0.0252 – 0.0376) | 4.81 (3.86 – 5.76) | Imputed |
| Niger | <0.0001 | <0.01 | Primary information available |
| Nigeria | 0.1375 (0.0008 – 0.7821) | 0.68 (<0.01 – 3.89) | Imputed |
| Niue | NA | NA | GDP missing [IMF/World Bank] |
| North Macedonia | 0.0319 (0.0148 – 0.475) | 15.34 (7.13 – 22.86) | Imputed |
| Norway | 2.1691 | 404.99 | Primary information available |
| Oman | 0.1327 (<0.0000 – 0.5899) | 30.82 (<0.00 – 136.99) | Imputed |
| Pakistan | 0.0489 | 0.24 | Primary information available |
| Palau | 0.0005 (<0.0000 – 0.0007) | 25.39 (0.02 – 38.87) | Imputed |
| Panama | 0.1354 (0.1087 – 0.1621) | 32.10 (25.76 – 38.43) | Imputed |
| Papua New Guinea | 0.0358 (0.0039 – 0.0829) | 4.17 (0.45 – 9.64) | Imputed |
| Paraguay | 0.0513 (0.0367 – 0.0790) | 7.18 (5.13 – 11.05) | Imputed |
| Peru | 0.2678 (0.2678 – 0.5766) | 8.24 (8.24 – 17.74) | Imputed |
| Philippines | 0.0165 | 0.15 | Primary information available |
| Poland | 1.7024 | 44.83 | Primary information available |
| Portugal | 0.8695 | 84.70 | Primary information available |
| Qatar | 0.2287 | 83.07 | Primary information available |
| Republic of Korea | 7.2547 | 139.93 | Primary information available |
| Republic of Moldova | 0.0051 | 1.43 | Primary information available |
| Romania | 0.3761 | 19.27 | Primary information available |
| Russian Federation | 0.8921 | 6.08 | Primary information available |
| Rwanda | 0.0035 (<0.0000 – 0.0122) | 0.28 (<0.00 – 0.99) | Imputed |
| Saint Kitts and Nevis | 0.0030 (<0.0000 – 0.0070) | 56.98 (<0.00 – 132.47) | Imputed |
| Saint Lucia | 0.0054 (0.0019 – 0.0090) | 29.98 (10.49 – 50.04) | Imputed |
| Saint Vincent and the Grenadines | 0.0026 (0.0009 – 0.0043) | 23.29 (8.15 - 38.87) | Imputed |
| Samoa | 0.0010 | 5.16 | Primary information available |
| San Marino | 0.0075 (0.0009 – 0.0154) | 222.24 (27.39 – 455.79) | Imputed |
| Sao Tome and Principe | 0.0001 (<0.0000 – 0.0008) | 0.61 (<0.00 – 3.49) | Imputed |
| Saudi Arabia | 1.2518 (<0.0000 – 5.5643) | 36.73 (<0.00 – 163.26) | Imputed |
| Senegal | 0.0044 | 0.27 | Primary information available |
| Serbia | 0.1100 (0.0511 – 0.1639) | 15.79 (7.34 – 23.53) | Imputed |
| Seychelles | 0.0013 | 13.61 | Primary information available |
| Sierra Leone | 0.0014 (<0.0000 – 0.0077) | 0.18 (<0.00 – 1.00) | Imputed |
| Singapore | 1.1604 (0.2325 –1.7986) | 204.66 (41.01 - 317.22) | Imputed |
| Slovakia | 0.3082 | 56.55 | Primary information available |
| Slovenia | 0.1215 | 58.73 | Primary information available |
| Solomon Islands | 0.0021 (0.0002 – 0.0048) | 3.26 (0.35 – 7.55) | Imputed |
| Somalia | 0.0026 (<0.0000 – 0.0089) | 0.17 (<0.00 – 0.59) | Imputed |
| South Africa | 2.2861 | 38.86 | Primary information available |
| South Sudan | 0.0011 (<0.0000 – 0.0040) | 0.09 (<0.00 – 0.30) | Imputed |
| Spain | 8.3063 | 178.03 | Primary information available |
| Sri Lanka | 0.0400 | 1.82 | Primary information available |
| Sudan | 0.0463 (<0.0000 – 0.1609) | 1.07 (<0.00 – 3.72) | Imputed |
| Suriname | 0.0095 | 15.90 | Primary information available |
| Sweden | 3.3758 | 327.04 | Primary information available |
| Switzerland | 4.2189 | 493.73 | Primary information available |
| Syrian Arab Republic | 0.0280 (<0.0000 – 0.1245) | 1.66 (<0.00 – 7.36) | Imputed |
| Tajikistan | 0.0306 | 3.29 | Primary information available |
| Thailand | 0.1917 | 2.82 | Primary information available |
| Timor-Leste | 0.0007 (<0.0001 – 0.0013) | 0.55 (<0.00 – 1.00) | Imputed |
| Togo | 0.0017 (<0.0000 – 0.0095) | 0.20 (<0.00 – 1.16) | Imputed |
| Tonga | 0.0017 | 16.62 | Primary information available |
| Trinidad and Tobago | 0.0737 | 53.40 | Primary information available |
| Tunisia | 0.0609 | 5.17 | Primary information available |
| Turkey | 0.8299 | 10.00 | Primary information available |
| Turkmenistan | 0.1348 (0.0163 – 0.4266) | 22.57 (2.72 – 71.43) | Imputed |
| Tuvalu | 0.0001 (<0.0000 – 0.0002) | 6.81 (<0.00 – 17.16) | Imputed |
| Uganda | 0.0124 | 0.31 | Primary information available |
| Ukraine | 0.0589 | 1.41 | Primary information available |
| United Arab Emirates | 0.6985 (<0.0000 – 3.1052) | 64.99 (<0.00 – 288.88) | Imputed |
| United Kingdom of Great Britain and Northern Ireland | 9.5769 | 143.22 | Primary information available |
| United Republic of Tanzania | 0.0001 | <0.01 | Primary information available |
| United States of America | 133.5058 | 405.46 | Primary information available |
| Uruguay | 0.1075 (0.0769 – 0.1655) | 30.56 (21.84 – 47.02) | Imputed |
| Uzbekistan | 0.2183 (0.0263 - 0.6910) | 6.61 (0.80 - 20.92) | Imputed |
| Vanuatu | 0.0001 | 0.51 | Primary information available |
| Venezuela (Bolivarian Republic of) | 0.4937 (0.3963 – 0.5911) | 17.93 (14.40 – 21.47) | Imputed |
| Viet Nam | 0.0570 (0.0002 – 0.1034) | 0.60 (<0.00 – 1.08) | Imputed |
| Yemen | 0.0098 | 0.31 | Primary information available |
| Zambia | 0.0008 | 0.04 | Primary information available |
| Zimbabwe | 0.0587 (0.0038 – 0.1137) | 3.94 (0.25 – 7.63) | Imputed |

Data source: Jevdjevic, M. and S. Listl, “Economic impacts of oral diseases in 2019 - data for 194 countries [database]” Heidelberg Open Research Data (heiDATA), 2022, https://doi.org/10.11588/data/JGJKK0.

**Table A.2:** Country-level indirect costs (productivity losses) due to five main oral conditions in 2019

|  | ***Five main oral conditions combined*** | | ***Caries in Deciduous Teeth*** | | ***Caries in Permanent Teeth*** | | ***Edentulism*** | | ***Periodontal diseases*** | | ***Other oral diseases*** | |
| --- | --- | --- | --- | --- | --- | --- | --- | --- | --- | --- | --- | --- |
| ***Location*** | **Total** | **Per capita** | **Total** | **Per capita** | **Total** | **Per capita** | **Total** | **Per capita** | **Total** | **Per capita** | **Total** | **Per capita** |
| Afghanistan | 0.04 | 1 | 0.0010 | 0.03 | 0.01 | 0.15 | 0.01 | 0.39 | 0.01 | 0.17 | 0.01 | 0.24 |
| Albania | 0.07 | 24 | 0.0003 | 0.11 | 0.01 | 1.96 | 0.04 | 14.38 | 0.01 | 4.26 | 0.01 | 3.03 |
| Algeria | 0.53 | 12 | 0.0051 | 0.12 | 0.05 | 1.22 | 0.26 | 5.98 | 0.13 | 2.93 | 0.09 | 2.10 |
| Andorra | 0.01 | 153 | 0.0000 | 0.44 | 0.00 | 11.43 | 0.01 | 77.83 | 0.00 | 41.77 | 0.00 | 21.55 |
| Angola | 0.20 | 6 | 0.0041 | 0.13 | 0.02 | 0.66 | 0.05 | 1.46 | 0.08 | 2.50 | 0.04 | 1.40 |
| Antigua and Barbuda | 0.01 | 63 | 0.0000 | 0.39 | 0.00 | 4.66 | 0.00 | 24.48 | 0.00 | 23.90 | 0.00 | 9.72 |
| Argentina | 1.46 | 33 | 0.0102 | 0.23 | 0.15 | 3.32 | 0.66 | 14.69 | 0.40 | 8.93 | 0.24 | 5.43 |
| Armenia | 0.07 | 22 | 0.0003 | 0.11 | 0.00 | 1.41 | 0.04 | 14.11 | 0.01 | 3.80 | 0.01 | 2.62 |
| Australia | 6.01 | 238 | 0.0238 | 0.94 | 0.37 | 14.66 | 3.80 | 150.67 | 1.05 | 41.55 | 0.77 | 30.50 |
| Austria | 2.20 | 246 | 0.0046 | 0.51 | 0.12 | 13.96 | 1.36 | 152.31 | 0.45 | 50.31 | 0.26 | 28.92 |
| Azerbaijan | 0.18 | 18 | 0.0013 | 0.12 | 0.01 | 1.47 | 0.10 | 9.74 | 0.04 | 3.76 | 0.03 | 2.72 |
| Bahamas | 0.05 | 123 | 0.0003 | 0.76 | 0.00 | 9.56 | 0.02 | 44.47 | 0.02 | 47.78 | 0.01 | 20.06 |
| Bahrain | 0.15 | 90 | 0.0007 | 0.41 | 0.01 | 8.38 | 0.06 | 37.03 | 0.05 | 29.23 | 0.02 | 15.12 |
| Bangladesh | 0.64 | 4 | 0.0083 | 0.05 | 0.08 | 0.50 | 0.06 | 0.38 | 0.33 | 2.01 | 0.16 | 0.97 |
| Barbados | 0.02 | 80 | 0.0001 | 0.30 | 0.00 | 4.81 | 0.01 | 38.32 | 0.01 | 26.44 | 0.00 | 10.42 |
| Belarus | 0.27 | 29 | 0.0013 | 0.14 | 0.02 | 2.30 | 0.15 | 15.62 | 0.06 | 6.79 | 0.04 | 3.86 |
| Belgium | 2.77 | 240 | 0.0065 | 0.57 | 0.13 | 11.67 | 1.62 | 140.03 | 0.71 | 61.83 | 0.30 | 26.06 |
| Belize | 0.00 | 12 | 0.0001 | 0.14 | 0.00 | 1.22 | 0.00 | 4.06 | 0.00 | 4.37 | 0.00 | 2.36 |
| Benin | 0.03 | 3 | 0.0006 | 0.05 | 0.00 | 0.29 | 0.01 | 0.43 | 0.01 | 1.19 | 0.01 | 0.57 |
| Bhutan | 0.01 | 8 | 0.0001 | 0.08 | 0.00 | 0.90 | 0.00 | 1.03 | 0.00 | 3.84 | 0.00 | 1.80 |
| Bolivia (Plurinational State of) | 0.15 | 13 | 0.0015 | 0.13 | 0.01 | 1.21 | 0.08 | 7.19 | 0.03 | 2.80 | 0.02 | 1.85 |
| Bosnia and Herzegovina | 0.10 | 30 | 0.0003 | 0.11 | 0.01 | 2.06 | 0.06 | 19.35 | 0.02 | 5.21 | 0.01 | 3.50 |
| Botswana | 0.06 | 26 | 0.0005 | 0.21 | 0.01 | 2.25 | 0.02 | 9.85 | 0.02 | 9.32 | 0.01 | 4.09 |
| Brazil | 8.29 | 39 | 0.0442 | 0.21 | 0.44 | 2.07 | 5.12 | 24.28 | 1.65 | 7.83 | 1.03 | 4.90 |
| Brunei Darussalam | 0.03 | 60 | 0.0003 | 0.67 | 0.00 | 7.71 | 0.01 | 15.36 | 0.01 | 20.19 | 0.01 | 16.43 |
| Bulgaria | 0.32 | 46 | 0.0011 | 0.16 | 0.02 | 3.49 | 0.21 | 29.31 | 0.05 | 7.52 | 0.04 | 5.68 |
| Burkina Faso | 0.03 | 2 | 0.0007 | 0.04 | 0.00 | 0.17 | 0.00 | 0.18 | 0.02 | 0.76 | 0.01 | 0.36 |
| Burundi | 0.01 | 0 | 0.0001 | 0.01 | 0.00 | 0.07 | 0.00 | 0.07 | 0.00 | 0.19 | 0.00 | 0.13 |
| Cabo Verde | 0.01 | 11 | 0.0000 | 0.09 | 0.00 | 0.98 | 0.00 | 2.05 | 0.00 | 5.48 | 0.00 | 1.92 |
| Cambodia | 0.05 | 3 | 0.0009 | 0.05 | 0.01 | 0.45 | 0.02 | 1.35 | 0.01 | 0.44 | 0.01 | 0.86 |
| Cameroon | 0.09 | 3 | 0.0016 | 0.06 | 0.01 | 0.39 | 0.01 | 0.55 | 0.04 | 1.65 | 0.02 | 0.74 |
| Canada | 6.56 | 175 | 0.0265 | 0.71 | 0.40 | 10.79 | 2.83 | 75.71 | 2.31 | 61.85 | 0.99 | 26.39 |
| Central African Republic | 0.01 | 1 | 0.0001 | 0.02 | 0.00 | 0.12 | 0.00 | 0.32 | 0.00 | 0.38 | 0.00 | 0.23 |
| Chad | 0.02 | 1 | 0.0005 | 0.03 | 0.00 | 0.16 | 0.00 | 0.28 | 0.01 | 0.58 | 0.00 | 0.31 |
| Chile | 1.01 | 53 | 0.0062 | 0.33 | 0.13 | 6.72 | 0.41 | 21.43 | 0.32 | 16.66 | 0.16 | 8.32 |
| China | 45.71 | 31 | 0.2731 | 0.19 | 3.45 | 2.36 | 18.59 | 12.68 | 14.46 | 9.87 | 8.94 | 6.10 |
| Colombia | 1.14 | 23 | 0.0058 | 0.12 | 0.13 | 2.57 | 0.43 | 8.45 | 0.40 | 8.02 | 0.18 | 3.54 |
| Comoros | 0.00 | 3 | 0.0000 | 0.04 | 0.00 | 0.39 | 0.00 | 0.64 | 0.00 | 1.38 | 0.00 | 0.70 |
| Congo | 0.01 | 1 | 0.0001 | 0.02 | 0.00 | 0.14 | 0.00 | 0.37 | 0.00 | 0.61 | 0.00 | 0.29 |
| Cook Islands* | NA | NA | NA | NA | NA | NA | NA | NA | NA | NA | NA | NA |
| Costa Rica | 0.23 | 46 | 0.0013 | 0.26 | 0.02 | 3.36 | 0.10 | 19.82 | 0.08 | 15.37 | 0.03 | 6.81 |
| Côte d'Ivoire | 0.14 | 5 | 0.0023 | 0.09 | 0.01 | 0.56 | 0.03 | 1.19 | 0.06 | 2.49 | 0.03 | 1.08 |
| Croatia | 0.32 | 77 | 0.0010 | 0.24 | 0.02 | 5.64 | 0.19 | 45.16 | 0.07 | 17.50 | 0.04 | 8.56 |
| Cuba | 0.47 | 41 | 0.0017 | 0.15 | 0.03 | 2.74 | 0.23 | 20.68 | 0.14 | 12.58 | 0.06 | 5.09 |
| Cyprus | 0.13 | 109 | 0.0004 | 0.32 | 0.01 | 8.76 | 0.07 | 58.11 | 0.03 | 25.86 | 0.02 | 16.25 |
| Czechia | 1.22 | 114 | 0.0045 | 0.42 | 0.09 | 8.11 | 0.74 | 69.42 | 0.25 | 22.93 | 0.14 | 13.50 |
| Democratic People's Republic of Korea | 0.04 | 2 | 0.0004 | 0.01 | 0.00 | 0.17 | 0.02 | 0.75 | 0.01 | 0.37 | 0.01 | 0.40 |
| Democratic Republic of the Congo | 0.10 | 1 | 0.0018 | 0.02 | 0.01 | 0.13 | 0.03 | 0.32 | 0.03 | 0.40 | 0.02 | 0.24 |
| Denmark | 1.68 | 291 | 0.0027 | 0.47 | 0.08 | 13.61 | 0.78 | 134.41 | 0.63 | 108.81 | 0.20 | 33.81 |
| Djibouti | 0.01 | 7 | 0.0001 | 0.11 | 0.00 | 0.83 | 0.00 | 0.89 | 0.00 | 3.25 | 0.00 | 1.59 |
| Dominica | 0.00 | 32 | 0.0000 | 0.12 | 0.00 | 2.30 | 0.00 | 14.15 | 0.00 | 10.40 | 0.00 | 4.60 |
| Dominican Republic | 0.30 | 28 | 0.0028 | 0.26 | 0.02 | 2.25 | 0.13 | 11.77 | 0.10 | 9.19 | 0.05 | 4.58 |
| Ecuador | 0.42 | 24 | 0.0033 | 0.19 | 0.04 | 2.15 | 0.22 | 12.73 | 0.10 | 5.60 | 0.06 | 3.30 |
| Egypt | 0.83 | 8 | 0.0108 | 0.11 | 0.08 | 0.82 | 0.38 | 3.82 | 0.19 | 1.91 | 0.16 | 1.58 |
| El Salvador | 0.09 | 14 | 0.0007 | 0.11 | 0.01 | 1.15 | 0.04 | 6.17 | 0.03 | 4.35 | 0.01 | 2.23 |
| Equatorial Guinea | 0.02 | 18 | 0.0004 | 0.29 | 0.00 | 2.00 | 0.00 | 3.59 | 0.01 | 8.26 | 0.01 | 4.18 |
| Eritrea | 0.00 | 1 | 0.0001 | 0.02 | 0.00 | 0.17 | 0.00 | 0.14 | 0.00 | 0.46 | 0.00 | 0.28 |
| Estonia | 0.15 | 110 | 0.0006 | 0.43 | 0.01 | 6.97 | 0.08 | 62.64 | 0.04 | 26.41 | 0.02 | 13.58 |
| Eswatini | 0.01 | 12 | 0.0002 | 0.15 | 0.00 | 1.40 | 0.00 | 4.16 | 0.00 | 4.08 | 0.00 | 2.06 |
| Ethiopia | 0.18 | 2 | 0.0047 | 0.04 | 0.02 | 0.22 | 0.02 | 0.22 | 0.08 | 0.68 | 0.05 | 0.46 |
| Fiji | 0.01 | 13 | 0.0002 | 0.20 | 0.00 | 1.89 | 0.01 | 6.80 | 0.00 | 0.95 | 0.00 | 3.22 |
| Finland | 1.38 | 250 | 0.0034 | 0.62 | 0.07 | 13.43 | 0.80 | 145.36 | 0.35 | 63.26 | 0.15 | 27.58 |
| France | 11.99 | 184 | 0.0336 | 0.52 | 0.93 | 14.25 | 7.14 | 109.64 | 2.36 | 36.26 | 1.53 | 23.49 |
| Gabon | 0.05 | 21 | 0.0005 | 0.24 | 0.00 | 1.99 | 0.01 | 5.04 | 0.02 | 9.59 | 0.01 | 4.16 |
| Gambia | 0.00 | 2 | 0.0001 | 0.03 | 0.00 | 0.20 | 0.00 | 0.31 | 0.00 | 0.97 | 0.00 | 0.37 |
| Georgia | 0.09 | 23 | 0.0004 | 0.11 | 0.01 | 1.52 | 0.06 | 14.55 | 0.02 | 4.28 | 0.01 | 2.69 |
| Germany | 19.40 | 232 | 0.0372 | 0.45 | 1.14 | 13.65 | 10.04 | 120.17 | 5.96 | 71.39 | 2.22 | 26.57 |
| Ghana | 0.16 | 5 | 0.0023 | 0.07 | 0.01 | 0.49 | 0.03 | 0.85 | 0.08 | 2.76 | 0.03 | 1.12 |
| Greece | 0.99 | 95 | 0.0023 | 0.22 | 0.07 | 6.82 | 0.62 | 59.06 | 0.18 | 17.21 | 0.12 | 11.19 |
| Grenada | 0.00 | 39 | 0.0000 | 0.22 | 0.00 | 3.04 | 0.00 | 15.51 | 0.00 | 13.99 | 0.00 | 6.18 |
| Guatemala | 0.22 | 13 | 0.0025 | 0.14 | 0.02 | 1.18 | 0.09 | 5.22 | 0.07 | 3.76 | 0.04 | 2.23 |
| Guinea | 0.03 | 2 | 0.0006 | 0.05 | 0.00 | 0.25 | 0.00 | 0.39 | 0.01 | 0.99 | 0.01 | 0.48 |
| Guinea-Bissau | 0.00 | 2 | 0.0001 | 0.03 | 0.00 | 0.24 | 0.00 | 0.30 | 0.00 | 0.83 | 0.00 | 0.39 |
| Guyana | 0.02 | 19 | 0.0002 | 0.20 | 0.00 | 1.79 | 0.01 | 7.17 | 0.01 | 6.79 | 0.00 | 3.52 |
| Haiti | 0.02 | 2 | 0.0003 | 0.03 | 0.00 | 0.21 | 0.01 | 0.66 | 0.01 | 0.61 | 0.00 | 0.39 |
| Honduras | 0.07 | 7 | 0.0008 | 0.09 | 0.01 | 0.70 | 0.03 | 2.70 | 0.02 | 2.16 | 0.01 | 1.30 |
| Hungary | 0.72 | 74 | 0.0025 | 0.26 | 0.06 | 5.82 | 0.49 | 50.96 | 0.08 | 7.78 | 0.09 | 9.56 |
| Iceland | 0.09 | 267 | 0.0003 | 0.98 | 0.01 | 25.19 | 0.05 | 139.90 | 0.02 | 62.77 | 0.01 | 37.97 |
| India | 7.25 | 5 | 0.0774 | 0.06 | 0.74 | 0.54 | 1.94 | 1.42 | 2.96 | 2.17 | 1.53 | 1.12 |
| Indonesia | 3.21 | 12 | 0.0309 | 0.11 | 0.30 | 1.11 | 1.16 | 4.29 | 1.09 | 4.04 | 0.63 | 2.32 |
| Iran (Islamic Republic of) | 1.80 | 22 | 0.0161 | 0.19 | 0.18 | 2.12 | 0.76 | 9.22 | 0.52 | 6.25 | 0.32 | 3.91 |
| Iraq | 0.58 | 15 | 0.0080 | 0.20 | 0.07 | 1.82 | 0.25 | 6.30 | 0.14 | 3.44 | 0.12 | 3.02 |
| Ireland | 1.44 | 296 | 0.0050 | 1.03 | 0.11 | 23.30 | 1.01 | 206.00 | 0.10 | 20.48 | 0.22 | 45.02 |
| Israel | 1.31 | 154 | 0.0099 | 1.16 | 0.12 | 13.52 | 0.72 | 84.25 | 0.27 | 31.93 | 0.20 | 23.21 |
| Italy | 9.54 | 158 | 0.0222 | 0.37 | 0.55 | 9.16 | 5.77 | 95.23 | 2.04 | 33.70 | 1.16 | 19.13 |
| Jamaica | 0.06 | 20 | 0.0004 | 0.13 | 0.00 | 1.62 | 0.02 | 8.46 | 0.02 | 6.78 | 0.01 | 3.21 |
| Japan | 23.66 | 187 | 0.0574 | 0.45 | 1.24 | 9.78 | 13.46 | 106.08 | 5.98 | 47.13 | 2.93 | 23.08 |
| Jordan | 0.12 | 12 | 0.0014 | 0.14 | 0.02 | 1.52 | 0.05 | 5.19 | 0.03 | 2.74 | 0.02 | 2.30 |
| Kazakhstan | 0.70 | 37 | 0.0059 | 0.32 | 0.05 | 2.71 | 0.41 | 22.08 | 0.13 | 7.07 | 0.10 | 5.32 |
| Kenya | 0.19 | 4 | 0.0043 | 0.08 | 0.02 | 0.42 | 0.02 | 0.46 | 0.09 | 1.68 | 0.05 | 1.00 |
| Kiribati | 0.00 | 3 | 0.0000 | 0.07 | 0.00 | 0.54 | 0.00 | 1.54 | 0.00 | 0.17 | 0.00 | 0.86 |
| Kuwait | 0.37 | 89 | 0.0025 | 0.59 | 0.04 | 8.84 | 0.14 | 34.18 | 0.12 | 28.94 | 0.07 | 16.39 |
| Kyrgyzstan | 0.03 | 4 | 0.0003 | 0.05 | 0.00 | 0.41 | 0.01 | 2.28 | 0.00 | 0.69 | 0.00 | 0.70 |
| Lao People's Democratic Republic | 0.03 | 4 | 0.0007 | 0.09 | 0.00 | 0.67 | 0.01 | 1.11 | 0.00 | 0.57 | 0.01 | 1.39 |
| Latvia | 0.15 | 77 | 0.0006 | 0.32 | 0.01 | 5.85 | 0.08 | 40.27 | 0.04 | 20.49 | 0.02 | 10.20 |
| Lebanon | 0.18 | 27 | 0.0015 | 0.22 | 0.02 | 2.37 | 0.10 | 13.93 | 0.04 | 6.08 | 0.03 | 4.15 |
| Lesotho | 0.01 | 4 | 0.0001 | 0.03 | 0.00 | 0.36 | 0.00 | 1.71 | 0.00 | 1.19 | 0.00 | 0.61 |
| Liberia | 0.01 | 2 | 0.0001 | 0.03 | 0.00 | 0.19 | 0.00 | 0.30 | 0.00 | 0.75 | 0.00 | 0.34 |
| Libya | 0.13 | 19 | 0.0009 | 0.13 | 0.01 | 2.01 | 0.06 | 8.90 | 0.03 | 4.75 | 0.02 | 3.37 |
| Lithuania | 0.23 | 84 | 0.0009 | 0.34 | 0.02 | 6.73 | 0.12 | 43.02 | 0.06 | 22.81 | 0.03 | 11.20 |
| Luxembourg | 0.30 | 495 | 0.0008 | 1.31 | 0.02 | 34.83 | 0.17 | 274.36 | 0.07 | 117.80 | 0.04 | 66.48 |
| Madagascar | 0.03 | 1 | 0.0006 | 0.02 | 0.00 | 0.17 | 0.00 | 0.13 | 0.01 | 0.42 | 0.01 | 0.26 |
| Malawi | 0.01 | 1 | 0.0003 | 0.02 | 0.00 | 0.10 | 0.00 | 0.11 | 0.00 | 0.27 | 0.00 | 0.18 |
| Malaysia | 0.90 | 28 | 0.0092 | 0.29 | 0.07 | 2.33 | 0.46 | 14.39 | 0.16 | 5.03 | 0.20 | 6.11 |
| Maldives | 0.02 | 29 | 0.0002 | 0.37 | 0.00 | 3.95 | 0.01 | 9.86 | 0.00 | 6.44 | 0.00 | 8.62 |
| Mali | 0.03 | 2 | 0.0008 | 0.04 | 0.00 | 0.22 | 0.00 | 0.24 | 0.02 | 0.84 | 0.01 | 0.42 |
| Malta | 0.06 | 128 | 0.0001 | 0.28 | 0.00 | 9.47 | 0.03 | 68.95 | 0.01 | 31.95 | 0.01 | 17.50 |
| Mauritania | 0.02 | 5 | 0.0003 | 0.07 | 0.00 | 0.48 | 0.00 | 1.09 | 0.01 | 2.08 | 0.00 | 0.92 |
| Mauritius | 0.05 | 39 | 0.0002 | 0.18 | 0.00 | 2.77 | 0.03 | 23.14 | 0.01 | 6.77 | 0.01 | 6.37 |
| Mexico | 4.52 | 35 | 0.0336 | 0.26 | 0.27 | 2.11 | 2.14 | 16.76 | 1.39 | 10.90 | 0.69 | 5.38 |
| Micronesia (Federated States of) | 0.00 | 8 | 0.0000 | 0.12 | 0.00 | 1.31 | 0.00 | 4.04 | 0.00 | 0.51 | 0.00 | 2.10 |
| Monaco* | NA | NA | NA | NA | NA | NA | NA | NA | NA | NA | NA | NA |
| Mongolia | 0.04 | 13 | 0.0005 | 0.15 | 0.00 | 1.22 | 0.02 | 6.58 | 0.01 | 2.74 | 0.01 | 2.27 |
| Montenegro | 0.02 | 40 | 0.0001 | 0.17 | 0.00 | 3.17 | 0.01 | 23.80 | 0.00 | 7.38 | 0.00 | 5.05 |
| Morocco | 0.45 | 12 | 0.0033 | 0.09 | 0.04 | 1.08 | 0.23 | 6.22 | 0.12 | 3.18 | 0.07 | 1.81 |
| Mozambique | 0.03 | 1 | 0.0007 | 0.02 | 0.00 | 0.13 | 0.00 | 0.11 | 0.01 | 0.33 | 0.01 | 0.23 |
| Myanmar | 0.12 | 2 | 0.0016 | 0.03 | 0.01 | 0.27 | 0.04 | 0.71 | 0.03 | 0.54 | 0.04 | 0.70 |
| Namibia | 0.04 | 18 | 0.0004 | 0.16 | 0.00 | 1.41 | 0.02 | 8.79 | 0.01 | 5.11 | 0.01 | 2.57 |
| Nauru | 0.00 | 13 | 0.0000 | 0.00 | 0.00 | 2.66 | 0.00 | 5.33 | 0.00 | 0.89 | 0.00 | 4.44 |
| Nepal | 0.05 | 2 | 0.0011 | 0.04 | 0.01 | 0.30 | 0.01 | 0.24 | 0.02 | 0.73 | 0.02 | 0.56 |
| Netherlands | 4.87 | 285 | 0.0107 | 0.63 | 0.24 | 14.27 | 3.25 | 189.88 | 0.85 | 49.91 | 0.51 | 30.04 |
| New Zealand | 1.02 | 213 | 0.0018 | 0.38 | 0.05 | 10.01 | 0.63 | 132.06 | 0.22 | 46.79 | 0.11 | 23.32 |
| Nicaragua | 0.04 | 6 | 0.0004 | 0.06 | 0.00 | 0.53 | 0.01 | 2.25 | 0.01 | 1.81 | 0.01 | 1.01 |
| Niger | 0.02 | 1 | 0.0008 | 0.03 | 0.00 | 0.13 | 0.00 | 0.18 | 0.01 | 0.44 | 0.01 | 0.25 |
| Nigeria | 0.83 | 4 | 0.0168 | 0.08 | 0.09 | 0.45 | 0.10 | 0.47 | 0.42 | 2.08 | 0.21 | 1.07 |
| Niue* | NA | NA | NA | NA | NA | NA | NA | NA | NA | NA | NA | NA |
| North Macedonia | 0.06 | 27 | 0.0002 | 0.11 | 0.00 | 2.26 | 0.03 | 16.36 | 0.01 | 5.05 | 0.01 | 3.55 |
| Norway | 1.83 | 340 | 0.0061 | 1.13 | 0.13 | 24.35 | 0.92 | 170.86 | 0.54 | 100.94 | 0.23 | 42.62 |
| Oman | 0.23 | 47 | 0.0024 | 0.47 | 0.03 | 5.67 | 0.08 | 15.82 | 0.07 | 14.61 | 0.05 | 10.01 |
| Pakistan | 0.62 | 3 | 0.0125 | 0.06 | 0.07 | 0.31 | 0.11 | 0.51 | 0.28 | 1.30 | 0.14 | 0.67 |
| Palau | 0.00 | 43 | 0.0000 | 0.00 | 0.00 | 5.35 | 0.00 | 24.98 | 0.00 | 3.57 | 0.00 | 8.92 |
| Panama | 0.23 | 55 | 0.0018 | 0.42 | 0.02 | 4.64 | 0.10 | 22.39 | 0.08 | 18.63 | 0.04 | 8.47 |
| Papua New Guinea | 0.04 | 5 | 0.0011 | 0.13 | 0.01 | 0.87 | 0.02 | 2.07 | 0.00 | 0.29 | 0.01 | 1.44 |
| Paraguay | 0.11 | 15 | 0.0011 | 0.15 | 0.01 | 1.26 | 0.05 | 7.38 | 0.03 | 3.70 | 0.02 | 2.77 |
| Peru | 1.01 | 31 | 0.0065 | 0.20 | 0.08 | 2.39 | 0.59 | 18.28 | 0.21 | 6.44 | 0.12 | 3.75 |
| Philippines | 0.82 | 8 | 0.0131 | 0.12 | 0.10 | 0.91 | 0.46 | 4.26 | 0.05 | 0.50 | 0.20 | 1.84 |
| Poland | 2.77 | 73 | 0.0099 | 0.26 | 0.16 | 4.33 | 1.53 | 40.43 | 0.72 | 19.00 | 0.34 | 9.10 |
| Portugal | 1.13 | 111 | 0.0021 | 0.21 | 0.06 | 5.97 | 0.69 | 67.35 | 0.24 | 23.90 | 0.14 | 13.35 |
| Qatar | 0.49 | 175 | 0.0030 | 1.05 | 0.06 | 19.97 | 0.15 | 52.76 | 0.18 | 64.88 | 0.10 | 36.00 |
| Republic of Korea | 4.99 | 97 | 0.0229 | 0.45 | 0.45 | 8.80 | 1.87 | 36.43 | 1.68 | 32.89 | 0.97 | 18.89 |
| Republic of Moldova | 0.08 | 19 | 0.0003 | 0.08 | 0.01 | 1.64 | 0.04 | 10.32 | 0.02 | 4.18 | 0.01 | 2.60 |
| Romania | 1.15 | 59 | 0.0043 | 0.22 | 0.10 | 4.96 | 0.67 | 34.49 | 0.24 | 12.27 | 0.14 | 7.43 |
| Russian Federation | 8.25 | 57 | 0.0361 | 0.25 | 0.51 | 3.50 | 4.99 | 34.20 | 1.74 | 11.92 | 0.98 | 6.70 |
| Rwanda | 0.02 | 2 | 0.0004 | 0.03 | 0.00 | 0.22 | 0.00 | 0.23 | 0.01 | 0.70 | 0.01 | 0.40 |
| Saint Kitts and Nevis | 0.00 | 68 | 0.0000 | 0.32 | 0.00 | 5.07 | 0.00 | 25.35 | 0.00 | 26.30 | 0.00 | 10.77 |
| Saint Lucia | 0.01 | 45 | 0.0000 | 0.20 | 0.00 | 3.24 | 0.00 | 18.86 | 0.00 | 15.82 | 0.00 | 6.69 |
| Saint Vincent and the Grenadines | 0.00 | 28 | 0.0000 | 0.20 | 0.00 | 2.44 | 0.00 | 11.48 | 0.00 | 9.37 | 0.00 | 4.16 |
| Samoa | 0.00 | 9 | 0.0000 | 0.16 | 0.00 | 1.32 | 0.00 | 4.38 | 0.00 | 0.54 | 0.00 | 2.14 |
| San Marino | 0.01 | 204 | 0.0000 | 0.00 | 0.00 | 14.39 | 0.00 | 113.66 | 0.00 | 48.92 | 0.00 | 27.34 |
| Sao Tome and Principe | 0.00 | 5 | 0.0000 | 0.07 | 0.00 | 0.52 | 0.00 | 0.85 | 0.00 | 2.44 | 0.00 | 0.99 |
| Saudi Arabia | 2.02 | 59 | 0.0198 | 0.58 | 0.29 | 8.36 | 0.81 | 23.57 | 0.45 | 13.21 | 0.45 | 13.12 |
| Senegal | 0.06 | 4 | 0.0009 | 0.05 | 0.01 | 0.47 | 0.01 | 0.87 | 0.03 | 1.57 | 0.01 | 0.70 |
| Serbia | 0.31 | 36 | 0.0012 | 0.14 | 0.02 | 2.82 | 0.20 | 22.34 | 0.05 | 6.21 | 0.04 | 4.22 |
| Seychelles | 0.00 | 41 | 0.0000 | 0.34 | 0.00 | 4.19 | 0.00 | 16.93 | 0.00 | 10.23 | 0.00 | 9.72 |
| Sierra Leone | 0.01 | 1 | 0.0002 | 0.02 | 0.00 | 0.15 | 0.00 | 0.22 | 0.00 | 0.26 | 0.00 | 0.26 |
| Singapore | 1.09 | 187 | 0.0056 | 0.97 | 0.10 | 17.42 | 0.37 | 63.76 | 0.38 | 66.23 | 0.23 | 38.93 |
| Slovakia | 0.46 | 85 | 0.0019 | 0.35 | 0.04 | 6.83 | 0.26 | 48.39 | 0.10 | 18.11 | 0.06 | 11.24 |
| Slovenia | 0.31 | 150 | 0.0009 | 0.45 | 0.02 | 8.38 | 0.20 | 94.21 | 0.07 | 31.98 | 0.03 | 14.95 |
| Solomon Islands | 0.00 | 4 | 0.0001 | 0.11 | 0.00 | 0.76 | 0.00 | 1.81 | 0.00 | 0.22 | 0.00 | 1.23 |
| Somalia | 0.01 | 1 | 0.0002 | 0.02 | 0.00 | 0.09 | 0.00 | 0.08 | 0.00 | 0.18 | 0.00 | 0.15 |
| South Africa | 1.23 | 21 | 0.0094 | 0.16 | 0.09 | 1.48 | 0.53 | 9.05 | 0.41 | 7.05 | 0.19 | 3.23 |
| South Sudan | 0.01 | 1 | 0.0002 | 0.02 | 0.00 | 0.09 | 0.00 | 0.07 | 0.00 | 0.30 | 0.00 | 0.17 |
| Spain | 4.50 | 96 | 0.0160 | 0.34 | 0.45 | 9.54 | 2.71 | 58.06 | 0.52 | 11.14 | 0.81 | 17.27 |
| Sri Lanka | 0.20 | 9 | 0.0019 | 0.09 | 0.02 | 0.97 | 0.08 | 3.89 | 0.05 | 2.14 | 0.05 | 2.14 |
| Sudan | 0.07 | 2 | 0.0013 | 0.03 | 0.01 | 0.24 | 0.03 | 0.75 | 0.01 | 0.34 | 0.02 | 0.38 |
| Suriname | 0.01 | 21 | 0.0001 | 0.16 | 0.00 | 1.23 | 0.00 | 8.43 | 0.00 | 7.41 | 0.00 | 3.37 |
| Sweden | 2.25 | 224 | 0.0084 | 0.84 | 0.17 | 17.07 | 1.22 | 121.41 | 0.56 | 56.20 | 0.29 | 28.93 |
| Switzerland | 2.83 | 329 | 0.0068 | 0.79 | 0.26 | 30.76 | 1.37 | 159.30 | 0.79 | 91.37 | 0.41 | 47.26 |
| Syrian Arab Republic | 0.05 | 3 | 0.0003 | 0.02 | 0.00 | 0.29 | 0.03 | 1.48 | 0.01 | 0.58 | 0.01 | 0.46 |
| Tajikistan | 0.02 | 2 | 0.0003 | 0.04 | 0.00 | 0.27 | 0.01 | 1.20 | 0.00 | 0.41 | 0.00 | 0.45 |
| Thailand | 1.81 | 26 | 0.0084 | 0.12 | 0.14 | 1.99 | 0.77 | 11.08 | 0.57 | 8.26 | 0.32 | 4.58 |
| Timor-Leste | 0.00 | 2 | 0.0001 | 0.05 | 0.00 | 0.38 | 0.00 | 0.81 | 0.00 | 0.35 | 0.00 | 0.62 |
| Togo | 0.01 | 2 | 0.0002 | 0.03 | 0.00 | 0.18 | 0.00 | 0.29 | 0.01 | 0.75 | 0.00 | 0.33 |
| Tonga | 0.00 | 11 | 0.0000 | 0.20 | 0.00 | 1.56 | 0.00 | 5.94 | 0.00 | 0.65 | 0.00 | 2.62 |
| Trinidad and Tobago | 0.09 | 68 | 0.0004 | 0.26 | 0.01 | 4.51 | 0.04 | 28.32 | 0.03 | 24.63 | 0.01 | 9.79 |
| Tunisia | 0.14 | 12 | 0.0009 | 0.08 | 0.01 | 1.04 | 0.07 | 6.40 | 0.03 | 2.87 | 0.02 | 1.84 |
| Turkey | 3.33 | 40 | 0.0148 | 0.18 | 0.27 | 3.27 | 1.85 | 22.15 | 0.76 | 9.15 | 0.43 | 5.17 |
| Turkmenistan | 0.14 | 24 | 0.0016 | 0.27 | 0.01 | 2.22 | 0.07 | 12.58 | 0.03 | 5.20 | 0.02 | 4.12 |
| Tuvalu | 0.00 | 10 | 0.0000 | 0.00 | 0.00 | 1.46 | 0.00 | 5.48 | 0.00 | 0.73 | 0.00 | 2.19 |
| Uganda | 0.06 | 1 | 0.0018 | 0.04 | 0.01 | 0.19 | 0.01 | 0.19 | 0.03 | 0.62 | 0.02 | 0.43 |
| Ukraine | 0.82 | 19 | 0.0028 | 0.06 | 0.05 | 1.12 | 0.50 | 11.36 | 0.17 | 3.83 | 0.10 | 2.17 |
| United Arab Emirates | 1.26 | 129 | 0.0060 | 0.61 | 0.12 | 12.65 | 0.43 | 44.07 | 0.47 | 48.00 | 0.23 | 23.25 |
| United Kingdom | 11.20 | 166 | 0.0235 | 0.35 | 0.80 | 11.87 | 7.16 | 106.03 | 1.61 | 23.77 | 1.61 | 23.90 |
| United Republic of Tanzania | 0.11 | 2 | 0.0015 | 0.03 | 0.02 | 0.29 | 0.02 | 0.27 | 0.05 | 0.86 | 0.03 | 0.52 |
| United States of America | 78.47 | 238 | 0.3873 | 1.18 | 4.77 | 14.48 | 43.80 | 133.09 | 17.50 | 53.17 | 12.02 | 36.54 |
| Uruguay | 0.24 | 68 | 0.0012 | 0.33 | 0.02 | 5.55 | 0.11 | 32.65 | 0.07 | 20.94 | 0.03 | 8.95 |
| Uzbekistan | 0.17 | 5 | 0.0021 | 0.06 | 0.02 | 0.53 | 0.08 | 2.55 | 0.03 | 1.01 | 0.03 | 0.93 |
| Vanuatu | 0.00 | 6 | 0.0000 | 0.14 | 0.00 | 0.98 | 0.00 | 2.87 | 0.00 | 0.32 | 0.00 | 1.59 |
| Venezuela (Bolivarian Republic of) | 0.24 | 8 | 0.0016 | 0.06 | 0.02 | 0.63 | 0.10 | 3.66 | 0.08 | 2.76 | 0.04 | 1.26 |
| Viet Nam | 0.71 | 7 | 0.0081 | 0.08 | 0.08 | 0.88 | 0.27 | 2.79 | 0.16 | 1.64 | 0.19 | 1.93 |
| Yemen | 0.05 | 2 | 0.0009 | 0.03 | 0.01 | 0.22 | 0.02 | 0.70 | 0.01 | 0.28 | 0.01 | 0.35 |
| Zambia | 0.04 | 2 | 0.0010 | 0.05 | 0.01 | 0.31 | 0.01 | 0.29 | 0.02 | 1.04 | 0.01 | 0.63 |
| Zimbabwe | 0.05 | 3 | 0.0006 | 0.04 | 0.00 | 0.30 | 0.02 | 1.27 | 0.02 | 1.06 | 0.01 | 0.62 |
| **Total** | **322.69** | **41.90** | **1.55** | **0.20** | **22.46** | **2.92** | **167.29** | **21.72** | **82.10** | **10.66** | **49.28** | **6.40** |

*GDP missing Data source: Jevdjevic, M. and S. Listl, “Economic impacts of oral diseases in 2019 - data for 194 countries [database],” Heidelberg Open Research Data (heiDATA), 2022, https://doi.org/10.11588/data/JGJKK0. Note: productivity losses are for five main oral conditions.
